# In Vitro Studies of the Effect of Oil Emulsions from Transgenic Flax Varieties on the Treatment of Wound Healing and Care of Human Skin with the Tendency to Inflammation

**DOI:** 10.3390/ijms26062544

**Published:** 2025-03-12

**Authors:** Izabela Jęśkowiak-Kossakowska, Tomasz Gębarowski, Katarzyna Skórkowska-Telichowska, Benita Wiatrak

**Affiliations:** 1 Department of Pharmacology, Faculty of Medicine, Wroclaw Medical University, Mikulicza-Radeckiego 2, 50-345 Wroclaw, Poland; benita.wiatrak@umw.edu.pl; 2 Department of Biostructure and Animal Physiology, The Wroclaw University of Environmental and Life Sciences, Kożuchowska 1/3, 51-631 Wroclaw, Poland; tomasz.gebarowski@upwr.edu.pl; 3 Department of Non-Surgical Clinical Sciences, Faculty of Medicine, Wrocław University of Science and Technology, Wybrzeże Wyspiańskiego 27, 50-370 Wroclaw, Poland; katarzyna.skorkowska-telichowska@pwr.edu.pl; 4 Department of Endocrinology, Jerzy Gromkowski Regional Specialist Hospital, Koszarowa 5, 51-149 Wroclaw, Poland

**Keywords:** transgenic flax, linseed oil, wound healing, atopic dermatitis, antioxidant effects

## Abstract

Excessive amounts of free-oxygen radicals produced during inflammation induce oxidative stress and lead to cell damage, thus delaying the transition of inflammation into the proliferation in the wound healing process. Oxidative stress on skin cells also plays an important role in the pathogenesis of inflammatory skin diseases. The aim of the planned in vitro studies was to assess the mechanisms of regenerative action and protection of cells against oxidative stress of three oil emulsions from transgenic (GMO) flax varieties M, B, and MB and a linseed emulsion from traditional NIKE linseed oil. Antioxidant and gene-protective properties were identified for the tested oil emulsions in a healthy cell model and in an in vitro model of cells under oxidative stress. The wound-healing regenerative potential of these linseed emulsions was also assessed in the proliferation, cell cycle, migration, and apoptosis and necrosis assays. The conducted research presented that the tested transgenic oil emulsions are safe for human skin because they do not induce the proliferation of skin cancer cells and, at the same time, induce the migration processes of normal human skin cells. Additionally, their use increases the ability to eliminate damaged cells. Transgenic linseed oils provide a gene-protective effect and an increased antioxidant effect, resulting in increased protection of skin cells against oxidative stress, which plays an important role in the pathogenesis of atopic dermatitis and psoriasis. Linen emulsion B has the best regenerative and protective properties against human epidermis cancer, which is probably due to the presence of an increased amount of stigmasterol in its composition along with the appropriate content of polyphenol compounds, as well as an increased amount of oleic and linoleic acids.

## 1. Introduction

Actually, the incidence of allergic and inflammatory skin diseases has been increasing [1,2], which may be caused by the action of various external stimuli on the skin, such as contact with irritants, UV radiation, repeated mechanical stimuli, allergens, and infections, as well as internal factors such as autoimmune reactions [3]. Often, inflammatory skin diseases lead to very painful and difficult-to-heal wounds due to vasculitis and/or vascular obstruction [4]. Chronic wounds mostly arise as a problem secondary to high-profile issues such as an ageing population, obesity, and diabetes [5]. Depending on the cytokine profile, various forms of dermatitis may occur [3,6]. The most prevalent inflammatory skin diseases include atopic dermatitis, psoriasis, lupus, lichen planus, systemic sclerosis, contact dermatitis, rosacea, and alopecia areata [7,8].

The pathophysiology of atopic dermatitis is still not fully understood, but it is believed to be driven by the disruption of the epidermal barrier, activation of specific T cell subsets, and dysbiosis of the commensal skin microbiome. The characteristic clinical symptom of the disease is eczema, which may be subacute (scattered, scaly, erythematous spots and oozing lumps) or chronic (flaking patches and plaques with pimples and lichenization). Histologically, acute lesions are characterized by sponginess/edema in between epidermal keratinocytes, mild or absent epidermal thickening, and infiltration inflammatory cells along with degranulated mast cells, while chronic changes show clear signs of epidermal thickening, less pronounced epidermas, and heavier mononuclear inflammatory infiltration [9]. Treatment of atopic dermatitis mainly involves the topical use of immunomodulators or steroids. The modification of hydrocortisone has led to the development of more potent topical steroids but with increased side effects due to skin atrophy. Other topical eczema medications that belong to calcineurin inhibitors, such as tacrolimus and pimecrolimus, may reduce the anticancer effects of the immune system and allow the cancer to grow unimpeded [10,11]. In developed countries, phytotherapy is considered an alternative to modern medicine. Ayurveda describes linseed as a substance with many beneficial properties for the skin, such as moisturizing, stabilizing skin pH, improving skin elasticity, and removing skin discoloration [12,13,14]. Linseed oil is one of the well-known vegetable oils that is a valuable source of omega-3 fatty acids, particularly α-linolenic acid (ALA) [14,15,16], which must be obtained from food because it is not synthesized in the human body [17,18]. ALA fights allergic diseases (allergic conjunctivitis, asthma, food allergy) and also inhibits inflammation in the skin in atopic dermatitis, acne, and psoriasis [19,20,21]. Furthermore, a study by a German–French group showed that daily supplementation with linseed oil improved skin appearance and led to reduced skin sensitivity, modulation of epidermal barrier function, and an inflammatory response to Nicotinate [22].

Additionally, severe injuries to the skin, such as burns, can lead to pathological wound-healing patterns, which are often characterized by dermal fibrosis, or excessive scarring, and chronic inflammation [23]. Currently, there are drugs available for patients with inflammatory skin diseases like atopic dermatitis, including those with a tendency to create wounds, which may lead to severe side effects, such as the induction of melanoma, because of the use of tacrolimus on the skin. Therefore, it is necessary to look for alternative substances of plant origin, which, in addition to effectiveness, will ensure the highest safety of use. In recent years, the therapeutic potential of medical plants traditionally used in dermatology has been explored, and some of them have been developed and approved as a drug or medical device for the treatment of skin disorders. Furthermore, an increasing number of herbal products have been developed in the field of medical cosmetics, often called “cosmeceuticals” [24].

The aim of the planned in vitro studies was to assess the mechanisms of protective effect on human skin and to determine the wound-healing potential of three oil emulsions from transgenic flax varieties M, B, and MB and one linseed emulsion from oil from the traditional NIKE flax variety against epidermal cell lines Normal Human Dermal Fibroblasts (NHDF), Human Dermal Microvascular Endothelial Cells (HMVEC), Normal Human Epidermal Keratinocytes (NHEK), tumor lines Human Skin Epidermoid Epithelial Cells (A431), and Human Monocytic Leukemia Cell Line (THP-1), as well as the model cell line Chinese Chamster Lung Fibroblasts (V79-4).

## 2. Results

### 2.1. Viability Assay

Cell viability assays are essentially used for screening the response of the cells against a drug or a chemical agent. In particular, the pharmaceutical industry widely uses viability assays to evaluate the influence of developed agents on the cells [25]. The effect of the tested oil emulsions derived from transgenic B, M, and MB and normal NIKE linseed oils on the viability of NHDF, HMVEC, NHEK, V79-4, THP1, and A431 cells was assessed in the MTT assay. Table 1 presented the IC_50_ values at which culture viability decreased by 50%. The biological effects presented in Table 1 indicate that all tested oil emulsions do not show cytotoxicity towards NHDF, HMVEC, and NHEK cell lines, while M and MB oil emulsions do not have cytotoxicity towards V79-4 and THP1 lines. All tested oil emulsions did not induce the proliferation of A431 cancer cells. Oil emulsion B is characterized by the strongest cytotoxicity properties against human epidermoid carcinoma of the A431 line, amounting to 1.90 ± 0.26 mg/mL, while the IC_50_ value of the reference drug doxorubicin against the A431 line is 1.049 ± 0.17 µg/mL. IC_50_ values were estimated by non-linear regression using the dependence of biological effects on the molar value of the concentration of compounds (four-parameter logistic model with a Hill coefficient).

### 2.2. Proliferation Assay

**Figure 1:**
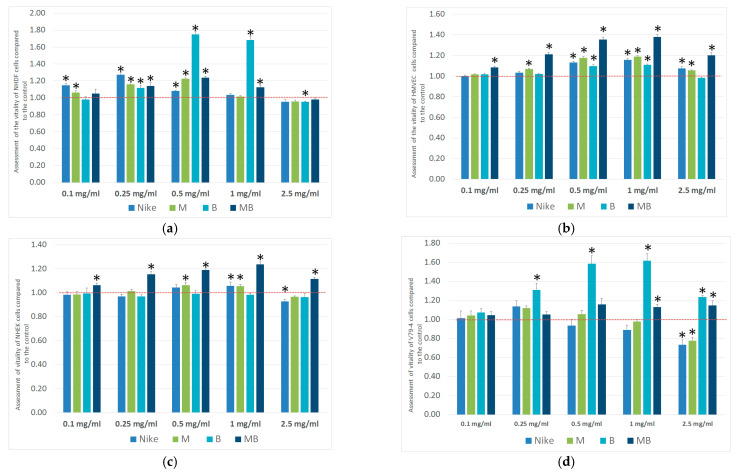
Results of SRB- (**a**) NHDF, (**b**) HMVEC, (**c**) NHEK, and (**d**) V79-4 after 48 h of incubation with test oil emulsions at five concentrations. Results are averages of five independent experiments ± SEM. Results are presented as the ratio of the value obtained in the test to the control. The result for the test oil emulsions was compared with the control (* *p* < 0.05) for NHDF 1 ± 0.025, HMVEC 1 ± 0.03, NHEK 1 ± 0.04, V79-4 1 ± 0.07.

The tested linseed oil emulsions caused increasing proliferation (in SRB assay) of cells such as NHDF, HMVEC, NHEK, and V79-4, as shown in Figure 1 below. There was a statistically significant increase in NHDF cell proliferation (Figure 1a) for oil emulsion B for most tested concentrations (0.25 mg/mL—1.12 ± 0.07, 0.5 mg/mL—1.75 ± 0.06, 1 mg/mL—1.68 ± 0.07, 2.5 mg/mL—0.95 ± 0.01). There was a statistically significant increase in the proliferation of NHDF cells for all oil emulsions for concentrations of 0.5 mg/mL and 1.0 mg/mL. For MB oil emulsion, there was a statistically significant increase in HMVEC cell proliferation at all tested concentrations (0.1 mg/mL—1.09 ± 0.02, 0.25 mg/mL—1.21 ± 0.07, 0.5 mg/mL—1.35 ± 0.05, 1 mg/mL—1.38 ± 0.05, 2.5 mg/mL—1.20 ± 0.05), and a significant increase at the highest level for the concentration of 1 mg/mL (Figure 1b). At oil emulsion concentrations of 0.5 mg/mL and 1 mg/mL, there was a statistically significant increase in the proliferation of HMVEC cells for all oil emulsions. Moreover, for MB oil emulsion, there was a statistically significant increase in NHEK cell proliferation at all tested concentrations (0.1 mg/mL—1.06 ± 0.02, 0.25 mg/mL—1.15 ± 0.02, 0.5 mg/mL—1.19 ± 0.01, 1 mg/mL—1.24 ± 0.02, 2.5 mg/mL—1.12 ± 0.01) and at the highest level for the concentration of 1 mg/mL (Figure 1c). Also, for **B** oil emulsion, there was a statistically significant increase in V79-4 cell proliferation for the most tested concentrations (0.25 mg/mL—1.31 ± 0.07, 0.5 mg/mL—1.58 ± 0.08, 1 mg/mL—1.62 ± 0.08, 2.5 mg/mL—1.24 ± 0.01)—Figure 1d.

### 2.3. Cell Cycle

**Figure 2:**
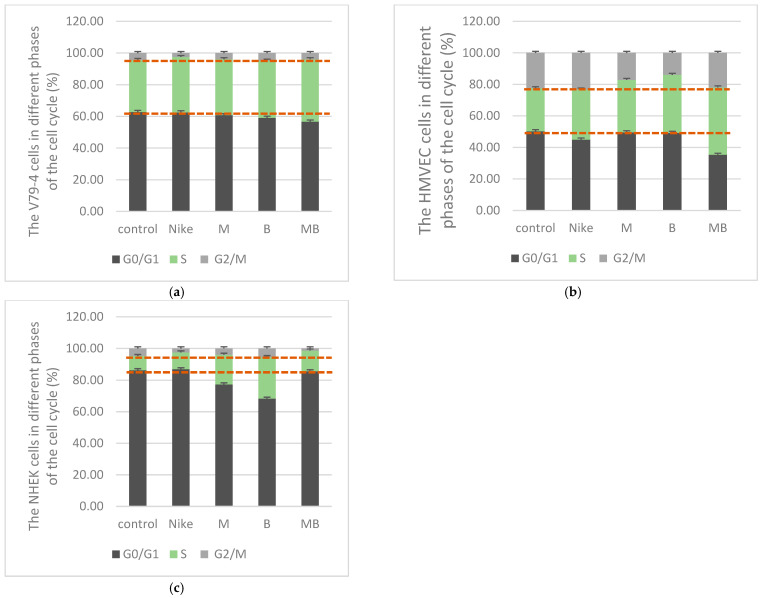
Cell cycle in (**a**) V79-4, (**b**) HMVEC, and (**c**) NHEK cells after 48 h of incubation with tested oil emulsions. The results are the means of five independent experiments.

Cell cycle studies were performed to assess the impact of the tested linseed emulsions on the course cell cycle and tissue regeneration [26]. In the cell cycle, the phase responsible for proliferation is the S phase. Twenty-four hours of incubation of V79-4 cells with the tested oil emulsions resulted in a tendency to increase the number of cells in the proliferative phase (S phase) for all tested oil emulsions, and to the greatest extent for the MB oil emulsion—39.33% (B—36.06%, M—35.31%, NIKE—34.93%)—Figure 2a compared to control (32.79%). After incubation of HMVEC cells with the tested oil emulsions, the number of cells in the S phase increased for all tested oil emulsions MB (42.69%), B (36.84%), M (33.31%), and NIKE (31.76%) compared to the control (27.27%)—Figure 2b. In the case of NHEK cells, incubation with the tested oil emulsions increased the number of cells in the proliferative phase (S phase) for tested oil emulsions B (26.22%), M (18.61%), MB (13.00%), and NIKE compared to the control (8.87%)—Figure 2c. The greatest number of NHEK cells occurred in the S phase for oil emulsion B but for HMVEC and V79-4 cells for oil emulsion MB. The tested emulsions were added at a concentration of 0.5 mg/mL.

Before the S phase of the cell cycle comes the G1 phase, in which incorrect genetic information can be replicated in the event of various DNA damage, which prevents the formation of defective cells in the next M phase. For all tested cell lines V79-4, HMVEC, and NHEK after incubation with linseed emulsions, the cells in the G0/G1 phase dominated, with the highest level determined for NHEK for NIKE oil emulsions (86.91%), and for transgenic oils lower than MB (85.63%), M (77.34%), and B (68.25%). The largest number in this phase was for HMVEC after incubation with M (49.55%) and B (49.21%) linseed emulsions, and V79-4 cells after incubation with NIKE (62.57%) and M (60.77%) oil emulsions.

### 2.4. Migration Assay

**Figure 3:**
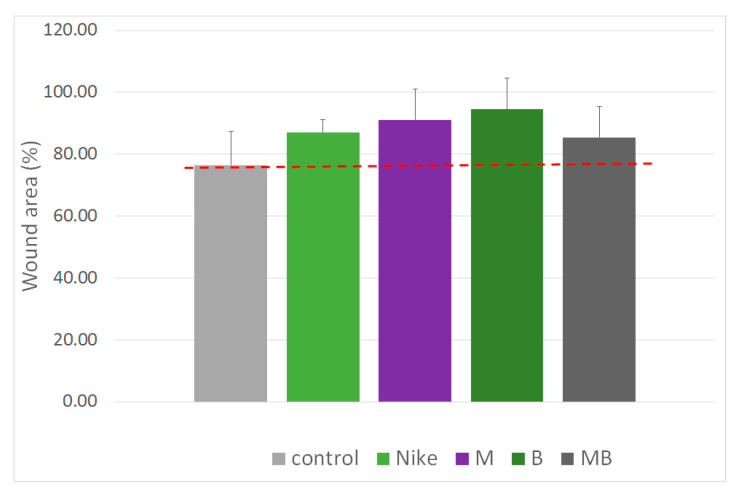
Migration percent of V79-4 cells after 20 h of incubation. The results are the means of five independent experiments.

The results of the cell migration test under the influence of the tested oil emulsions, allow for the assessment of their potential applications, e.g., in the regeneration of skin tissues [27]. A cell migration assay was performed against V79-4 cells to assess whether the tested oil emulsions induce wound healing. The seeded cells were incubated until a monolayer was formed over the entire surface of the well. Then, a scratch test was performed by making a scratch in the monolayer and measuring its width. The tested emulsions were added at a concentration of 0.25 mg/mL. Cell migration of the created cracks was observed for all oil emulsions, with the highest degree of cell migration occurring after 20 h for linseed oil emulsion B (94.50%). However, the cell migration for oil emulsion M was on the level of 91.09%; for NIKE oil emulsion—86.95%; and for MB oil emulsion—85.47%. The cell migration in the control was 76.41% (Figure 3).

### 2.5. Apoptotic and Necrotic Cells

**Figure 4:**
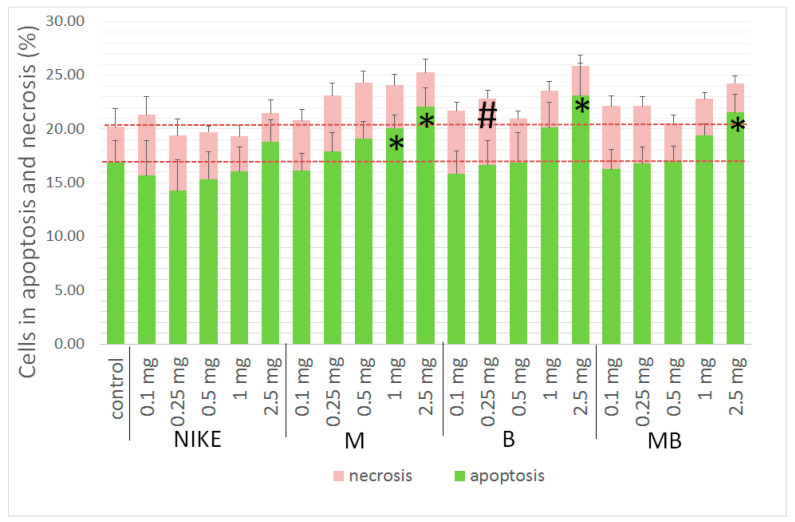
Apoptosis and necrosis of V79-4 cells after 24 h of incubation with test oils at five concentrations and then damaged by H_2_O_2_ treatment (100 μM, 30 min, 4 °C). Results are presented as a percentage of apoptotic and necrotic cells. Results are averages of five independent experiments. Statistical significance of differences between the results for the tested oil emulsions compared to the control for apoptosis (* *p* < 0.05) and the control for necrosis (# *p* < 0.05).

An important step in wound repair is the elimination of cell death induced by the inflammatory environment [28]. The biogenerated apoptosis, in oxygen-related environmental stress, can promote vascularization [29]. There was no effect after 24 h of incubation of V79-4 cells uninjured with H_2_O_2_ and incubated only with the test oil emulsions. A statistically significant increase in the level of apoptosis occurred for linseed emulsion M at two concentrations: 1 mg/mL (20.07%) and 2.5 mg/mL (22.06%), and for linseed emulsion B (23.11%) and MB (21.52%) at a concentration of 2.5 mg/mL was indicated after 24 h incubation V79-4 cells with tested oil emulsions at five concentrations and then damaged by H_2_O_2_ treatment (100 μM, 30 min, 4 °C). Necrosis is statistically significant only for emulsion B (6.13%) with a concentration of 0.25 mg/mL. The lowest number of cells in the apoptosis phase was observed in cultures treated with NIKE oil emulsion. The process of apoptosis in the tested V79-4 cells clearly dominates over the process of necrosis (Figure 4).

### 2.6. Evaluation of the Intracellular Free Radical Level

**Figure 5:**
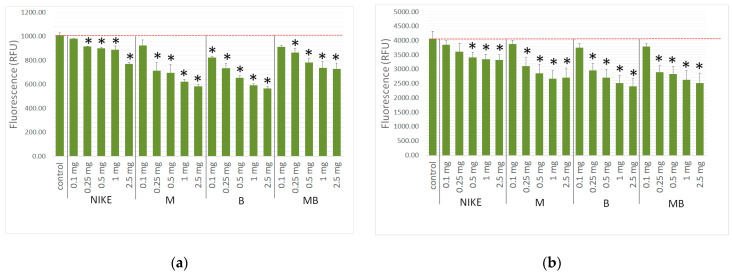
The ROS levels in V79-4 cells were incubated in the presence of tested linseed oil emulsions: untreated (**a**,**b**) treated with H_2_O_2_ (100 μM, 30 min). Results are presented as RFU (relative fluorescent units) of fluorescence level. Statistical significance was calculated in comparison to control (* *p* < 0.05).

Antioxidant therapies have been considered promising strategies in chronic wound healing due to their central role in downregulating ROS and suppressing oxidative stress. There is strong evidence that shows the beneficial roles of naturally derived antioxidants in wound-healing applications by efficiently promoting different phases of wound healing. There is an urgent need to develop advanced therapies to treat oxidative stress and restore redox hemostasis in skin regeneration [30,31,32]. In both research systems (untreated and treated H_2_O_2_ V79-4 cells), all linseed emulsions reduced the level of free radicals compared to the control. Also, in two research systems, oil emulsions from transgenic flax varieties caused the most reduction of the level of free radicals. A statistically significant reduction in the level of free radicals after the administration of oil emulsions to V79-4 cells in the entire range of tested concentrations 0.25 mg/mL–2.5 mg/mL was obtained for oil emulsions B and MB, and for M and NIKE. The greatest reduction in the level of free radicals occurred after treatment of V79-4 cells (untreated H_2_O_2_) was for oil emulsion B—0.25 mg/mL: 736.1 RFU ± 79.3; 0.5 mg/mL: 655.7 RFU ± 33.3; 1 mg/mL: 592.7 RFU ± 27.3; 2.5 mg/mL: 565.3 RFU ± 33.1 (Figure 5a).

A statistically significant reduction in the level of free radicals after treatment of V79-4 cells with H_2_O_2_ (100 µM for 30 min, at 4 °C) was obtained for the M, B, and MB oil emulsions in the concentration range 0.25 mg/mL–2.5 mg/mL, and for the NIKE oil emulsion 0.5 mg/mL–2.5 mg/mL. Also, the most statistically significant decreased levels of free radicals was for oil emulsion B after treatment of V79-4 cells (treated H_2_O_2_)—0.1 mg/mL: 3749.74 RFU ±296; 0.25 mg/mL: 2962 RFU ± 480; 0.5 mg/mL: 2709 RFU ± 554; 1 mg/mL: 2511 RFU ±532; 2.5 mg/mL: 2409 RFU ± 519 (Figure 5b).

### 2.7. Genotoxicity Assessment

**Figure 6:**
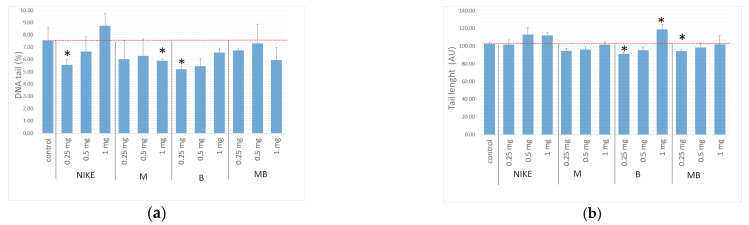
Level of DNA damage in V79-4 cells cultured for 24 h in the presence of the tested linseed oils at three different concentrations: (**a**) amount of DNA in the comet tail; (**b**) tail length. Results are averages of five independent repetitions. Statistical significance of differences between the results for the tested flaxseed oils compared to the control was marked (* *p* < 0.05).

**Figure 7:**
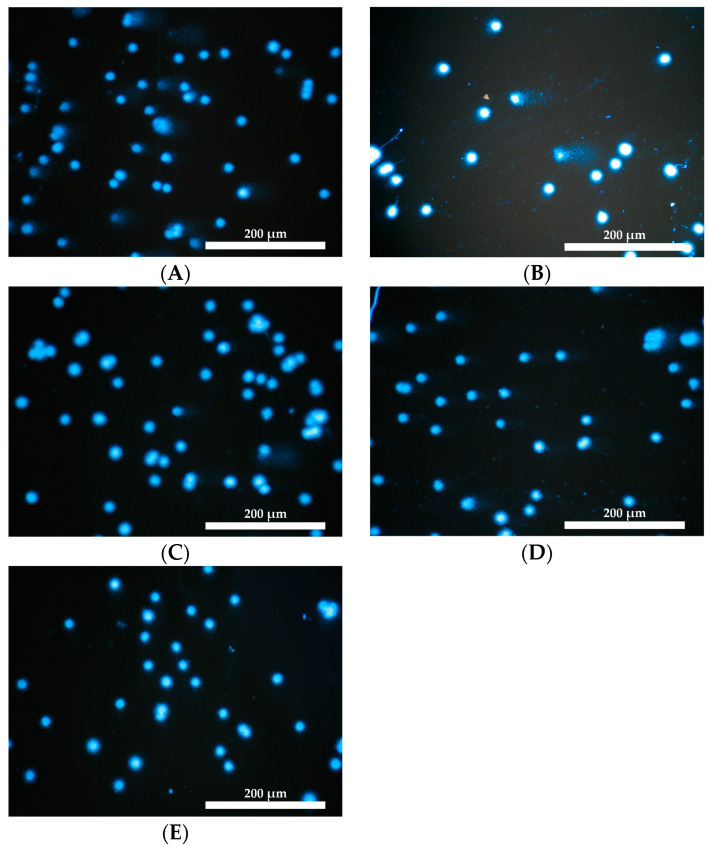
Protective effect of oil emulsions on the amount of DNA damage to cells damaged in the comet assay. Magnification 20×, DNA staining with DAPI dye. (**A**) control; (**B**) NIKE; (**C**) M; (**D**) B, and (**E**) MB.

It is crucial to examine whether the tested linseed emulsions being GMO products do not have genotoxic effects, which are closely related to diseases such as cancer [33]. Genome instability accompanies age-related diseases, including cancer, heart failure, chronic kidney disease, type 2 diabetes, chronic obstructive pulmonary disease, osteoporosis, stroke, Parkinson’s disease, Alzheimer’s disease, atherosclerosis, and sarcopenia [34]. The tested linseed oils presented safety when administered to intact V79-4 cells (Figure 6). Only in the case of oil emulsion B at the concentration of 1 mg/mL was there a statistically significant increase in tail length. At the same time, for oil emulsion B at the lowest concentration of 0.25 mg/mL, there was a statistically significant shortening of the tail (Figure 6b) and a reduction in the amount of DNA in the tail (Figure 6a), which proves the protective effect of oil emulsion B on undamaged V79-4 cells at the lowest concentration tested. A statistically significant shortening of the tail occurred for the MB oil emulsion at the lowest tested concentration of 0.25 mg/mL against intact V79-4 cells, and a statistically significant reduction in the amount of DNA in the tail occurred in the lowest concentration of the NIKE oil emulsion and the highest concentration of the M oil emulsion (1 mg/mL). All the tested oil emulsions did not indicate any genotoxic effect (Figure 7A–D).

**Figure 8:**
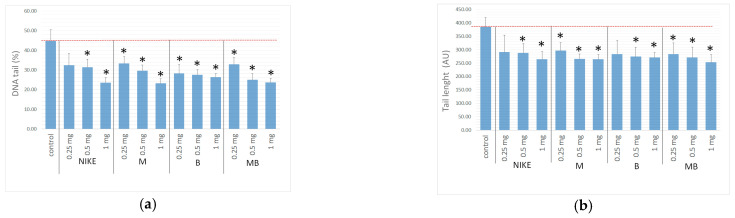
Level of DNA damage in V79-4 cells cultured for 24 h in the presence of tested linseed oils at three different concentrations and then damaged by H_2_O_2_ treatment (100 μM, 30 min, 4 °C): (**a**) amount of DNA in the comet tail (**b**) tail length. Results are averages of five independent studies. Statistical significance of differences between the results for the tested linseed oils compared to the control (* *p* < 0.05).

**Figure 9:**
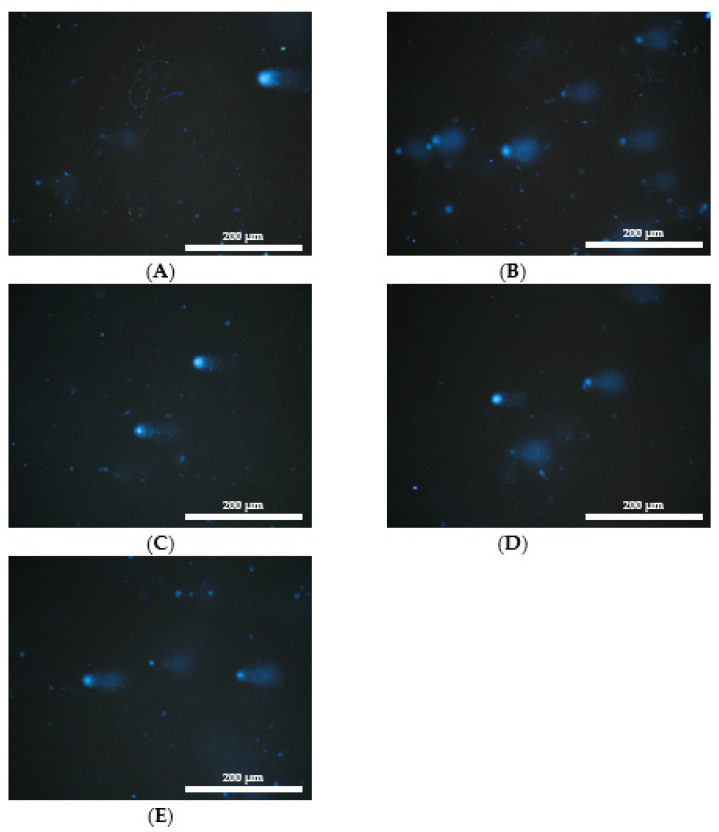
Protective effect on the amount of DNA damage and length of the tail to cells damaged by 100 µM of H_2_O_2_ (20 min, 4 °C) in the comet assay. Magnification 20×, DNA staining with DAPI dye. (**A**) control H_2_O_2_ 100 µM, 20 min, 4 °C; (**B**) NIKE; (**C**) M; (**D**) B; and (**E**) MB.

The tested linseed emulsions had a gene-protective effect towards damaged V79-4 cells (Figure 8 and Figure 9). A statistically significant reduction in the amount of DNA in the tail and tail length (except for oil emulsion B at a concentration of 0.25 mg/mL) occurs for all tested concentrations of all emulsions containing oils from transgenic flax varieties. For the emulsion with traditional NIKE oil, a statistically significant reduction in the amount of DNA in the tail and tail length occurred for two higher concentrations (0.5 mg/mL, 1 mg/mL) Figure 8.

## 3. Materials and Methods

### 3.1. Material

The material for the study consisted of flaxseed oil cold-pressed in a hydraulic screw-press at room temperature from the flaxseed variety Nike and 2 transgenic lines, M and B [35,36]. A 1:1 (*v*:*v*) mixture of M and B oil was created, which was called MB oil. Chemical characteristics such as fatty acid composition, phytosterol composition and content, composition of tocopherols, and 8-plastochromanol content, carotenoid composition and content, polyphenolic compounds and composition of studied NIKE, B, M and MB oils have already been published. In this study, linseed oils from the same harvests and the same batch of prepared emulsions NIKE, B, M, and MB were used [35].

Linseed oil emulsions were prepared for in vitro tests according to the protocol developed by Skorkowska–Telichowska et al. [37], as well as Gębarowski et al. [35]. The oils were introduced into the medium in the form of an emulsion (2.5 mL of oil, 0.5 mL of Tween 80 with 1 g of soy lecithin, and 5 mL of aqueous phase containing glycerol 25% by volume). Linseed oils were tested at final concentrations of 0.1 mg, 0.25 mg, 0.5 mg, 1.0 mg, and 2.5 mg per 1 mL of medium at room temperature and under sterile conditions. The control is an oil-free emulsion. The emulsions added to the culture were stable and did not separate [35].

### 3.2. In Vitro Studies

#### 3.2.1. Cell Line and Conditions

The viability test was performed on A431, NHDF, HMVEC, NHEK, THP-1, and V79-4 cell lines. The A-431 cell line serves as an invaluable cellular model for human epidermoid carcinoma, facilitating a deeper understanding of EGFR signaling, tumor biology, and the development of therapeutic interventions to combat epidermoid carcinoma and other related cancers. HMVEC secretes a mammary-derived growth inhibitor to modulate endothelial cell proliferation and differentiation of the mammary gland. HMVEC also regulates trafficking of lymphocytes and the inflammatory response and are, thus, related to mastitis. THP-1 cells are used to study various aspects of monocyte/macrophage biology, including differentiation, activation, response to infection, and the role of macrophages in disease processes such as cancer and inflammation. The V79-4 cell line is particularly known for its use in genetic toxicology research, including tests examining mutagenesis, cytotoxicity, and DNA repair mechanisms.

NHDF can be used for wound-healing studies and dermatological research to investigate diseases like scleroderma, fibrosarcoma, fibrosis, xeroderma pigmentosum, and histiocytoma. Moreover, fibroblasts are important for cancer research, tissue regeneration, and tissue engineering studies. NHEK is the major cell type in the epidermis, making up about 90% of the cells. Epidermal keratinocytes originate in the stratum basale and move up through the layers of the epidermis. During this movement, they undergo gradual differentiation and morphology changes until they reach the stratum corneum, where they form a layer of nucleus-free, flat, and highly keratinized squamous cells. This layer forms an effective barrier to the entry of infectious agents into the body and minimizes moisture loss. Keratinocytes are also able to produce a variety of cytokines, growth factors, interleukins, and complement factors. Therefore, keratinocytes are important for wound healing, inflammation, and immune response. The tests were performed in five independent replicates.

Cell lines were purchased from Lonza (Basel, Switzerland): Human Normal Dermal Fibroblast (NHDF), Human Dermal Microvascular Endothelial Cells—Adult (HMVEC). Normal Human Epidermal Keratinocytes (NHEK) were obtained from PromoCell (Heidelberg, Germany). Monocytic Cell Line (THP-1) and Human Skin Epidermoid Epithelial Cells A431 were obtained from ATCC (Manassas, VA, USA). The Chinese Hamster Lung Fibroblasts (V79-4 cells) were obtained from the Institute of Immunology and Experimental Therapy of the Polish Academy of Sciences in Wroclaw, Poland.

All cells were grown at 37 °C, in 5% CO_2_, 95% humidity with morphology evaluation. If confluence exceeds 70%, cell cultures were passaged using the TrypLe (London, UK) solution, counted using a Brucker chamber, and seeded in 96-well plates for assay. If the confluence was less than 70%, the medium was replaced with fresh. NHDF was cultured in Dulbecco’s modified Eagle medium (DMEM) without phenol red and NHEK was grown in the KBM–Gold medium (Keratinocyte Cell Basal Medium). A431 was incubated in Dulbecco’s modified Eagle medium (DMEM). THP-1 cells were incubated in an RPMI-1640 medium. HMVEC was grown in an EGM medium, which was enriched according to Lonza’s procedure. Other media were supplemented with 10% fetal bovine serum (FBS), penicillin (10,000 U/mL), streptomycin (10 mg/mL), and L-glutamine (200 mM). V79-4 was grown in EMEM with 2 mM L-glutamine, 10% FBS, and antibiotics: 100 U/mL penicillin and 0.1 mg/mL streptomycin.

#### 3.2.2. Viability Assay

In the MTT assay, detached cells were observed after 24 h of incubation with oil emulsions, and A431, NHDF, HMVEC, NHEK, THP-1, and V79-4 cell lines, the supernatant was removed and the solvents were rinsed with PBS. Additionally, the reference drug for the A431 line was doxorubicin.

The tetrazolium salt at a concentration of 1 mg/mL was added to each well. For the next 2 h, the culture was incubated in a CO_2_ incubator. The resulting formazan crystals were dissolved in isopropanol with shaking for 30 min. Spectrophotometric measurement at 570 nm used a Multiskan GO Thermo Fisher Scientific (Waltham, MA, USA) microplate reader.

#### 3.2.3. Proliferation Assay

Proliferation evaluation was performed using the SRB dye (sulforhodamine B), which binds to cellular proteins and provides information about protein content. After 24 h of cell adhesion, the assay was performed. One culture plate was left as a control, which was fixed with a cold TCA (trichloroacetic acid) solution at a final concentration of 10% *w*/*v* for 1 h at 4–8 °C. The other plates were cultured with the tested emulsion at 37 °C in a 5% CO_2_, 95% humidity environment. After the incubation period, all plates were fixed with a cold TCA solution. Plates were cleaned four times with running water and dried at room temperature (RT). A 0.4% SRB solution in 1% *v*/*v* acetic acid was added to the fixed cultures for 30 min. The excess stain was washed five times with a 1% acetic acid solution. The SRB dye was dissolved using 10 mM of Trizma base for 30 min with shaking. The absorbance was measured at 540 nm using a Multiscan Go microplate reader. All reagents used in the study were purchased Sigma–Aldrich (Darmstadt, Germany).

#### 3.2.4. Cell Cycle

After 24 h of incubation of V79-4, NHEK, and HMVEC cells with linseed emulsions, their effect on the cell cycle was assessed at a concentration of 0.5 mg/mL. After incubation, the cells were separated and centrifuged, and then the pellet was fixed with cold ethanol (70%) at room temperature for 10 min and centrifuged again at 600× *g* for 5 min. The cell pellet was suspended in propidium iodide and left in the dark for 10 min. Samples were transferred to chips and analyzed on an image-based cytometer Arthur^TM^ (NanoEnTek Inc., Seoul, Republic of Korea).

#### 3.2.5. Scratch Assay

The migration test was performed according to the procedure described by Gębarowski [35]. The tested emulsions were added at a concentration of 0.25 mg/mL. The SPLScar kit (SPL Life Sciences, Korea) was used to perform the scratch test. After seeding the cells, they were incubated until a monolayer was formed. A scratch of the same thickness and location was made in each well of the cell-covered plate using SPLScar, and then photos were taken. Freshly prepared linseed emulsions were added to the surfaces of monolayers with a scratch. Culture plates were incubated for 24 h. Microphotographs were taken using Julia’s microscope (NanoEnTek Inc., Seoul, Republic of Korea) and analyzed. The equipment took photos of the degree of fouling during 24 h of incubation in the incubator.

#### 3.2.6. Apoptotic and Necrotic Assay

The administration of oil emulsions to V79-4 cells was in the entire range of tested concentrations of 0.25 mg/mL–2.5 mg/mL. After 24 h of incubation, V79-4 cells were separated from the culture plates, centrifuged, suspended in binding buffer, and stained with a mixture of fluorochromes (Alexa Fluor 488 Annexin V and PI fluorescent dyes). Samples were measured after 15 min of incubation at room temperature in the dark on an image-based cytometer Arthur (NanoEnTek Inc.). Fluorescence, granulation, and size were measured for 40,000–10,000 cells. The percentage of live, apoptotic, and necrotic cells was determined from the prepared scatter plots.

#### 3.2.7. Evaluation of the Intracellular Free Radical Level

The administration of oil emulsions to V79-4 cells was in the entire range of tested concentrations of 0.25 mg/mL–2.5 mg/mL. Thirty thousand cells were seeded into 96-well plates, and the culture plates were incubated at 37 °C in 5% CO_2_ at 95% humidity overnight. After 24 h, the supernatant was removed. Tested oil emulsions for cell culture were added and incubated in the same conditions for 1 h. Twenty-five μM of freshly prepared DCF-DA solution were added to the cells in PBS for 20 min at 37 °C in 5% CO_2_ in the dark. Some of the tested cells in the system for determining the influence of antioxidant properties of the tested oil emulsions on damaged cells were washed twice with PBS, and H_2_O_2_ (100 μM) was added for 30 min. Dichlorodihydrofluorescein fluorescence (λex = 485 nm, λem = 535 nm) was read using a Victor 2 microspecttrophotometer (PerkinElmer, Waltham, MA, USA).

#### 3.2.8. Genotoxicity Assessment

**Scheme 1:**
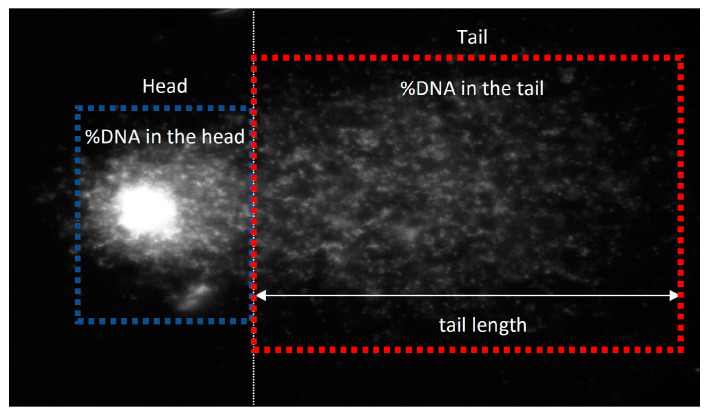
The principle of measuring tail and head length in the comet assay.

The administration of oil emulsions to V79-4 cells was in the entire range of tested concentrations of 0.25 mg/mL–2.5 mg/mL. The comet assay was performed according to the procedure of Collins [38]. Cells were cultured in the presence of oil emulsions for 48 h. V79-4 cells were separated using trypsin/EDTA from the culture vessel, centrifuged, and washed in PBS before being cooled to 4 °C without Ca^2+^ or Mg^2+^ ions. In an ice-water bath (4 °C) for 30 min, cells were incubated with PBS and supplemented with H_2_O_2_ (100 μM). After incubation, the cells were dissolved in an excess volume of chilled PBS and centrifuged, and the cell pellet was resuspended in cold PBS containing Ca^2+^ and Mg^2+^ ions. Cells in suspension were mixed with an equal volume of 1% low melting point agarose (Sigma VII) previously heated in a water bath at 37 °C. Suspensions were placed on slides pre-coated with 0.5% plain agarose (Sigma I-A type). The slides were carefully immersed in cold (4 °C) lysis solution (2.5 M NaCl, 100 mM EDTA, 10 mM of Tris, pH 10, 1% Triton X-100, and 10% DMSO) and incubated in the dark at 4 °C overnight. The slides were washed (five times for 5 min each) with alkaline electrophoresis buffer (300 mM of NaOH and 1 mM of EDTA, pH 13) and then placed in a horizontal gel electrophoresis device filled with freshly prepared alkaline electrophoresis buffer. The slides were exposed to alkali for 45 min at 4 °C. Electrophoresis (1.2 V/cm, 300 mA) was performed for 20 min at 4–6 °C, and the slides were washed with neutralizing buffer (0.4 M Tris, pH 7.5) four times for 5 min each. Finally, the slides were immersed in fluorescent dye (DAPI, 1 μg/mL) and stained overnight in the refrigerator. All steps were performed in low light. Sections were analyzed using a Nikon Eclipse E600 microscope in the Comet IV program. The principle of measuring tail and head length in the comet test is shown in Scheme 1.

### 3.3. Statistical Analysis

All assays were performed in five independent replicates. Statistical calculations were performed using parametric tests (normal distribution and equal variance of the obtained results). Statistical significance was calculated using Tukey’s post-hoc test using GraphPad Prism Version 6.05 (GraphPad Software, La Jolla, CA, USA). The significance point was set at * *p* < 0.05.

## 4. Discussion

Three major goals of wound care are keeping wounds clean, promoting wound healing, and minimizing scarring and discomfort [39,40]. Some elements, such as persistent wounds, infections, sluggish tissue regeneration, and poor wound closure, can seriously hamper healing and have negative effects. The development of innovative treatment approaches can effectively facilitate the wound-healing process, improve patient well-being, and reduce the societal burden [41,42].

Oils rich in omega-3 fatty acids (polyunsaturated fatty acids—PUFA), such as linseed oil, have become a trend due to their clinically proven health benefits [43]. Linseed oil, because of its anti-allergic properties and high content of phytochemicals, can be used as a base for medicinal and cosmetic substances [12] while protecting the skin against inflammation [12,44], improving its elasticity, and soothing irritations, as well as healing scars [45]. In addition, it lightens scars due to its high content of antioxidants and PUFA [12,46]. Also, linseed oil is used to heal burn wounds [14,45,47,48]. The linseed oils tested in this work also contain significant amounts of PUFA.

In linseed oil, although the level of phenols is favorable, it is quite low, therefore increasing the antioxidant properties of linseed oil and its stability. Three types of GMO flax plants were obtained, i.e., W92, W86, and GT, with an increased content of phenolic ingredients in flax seeds. As a result, oil from transgenic types of flax contained an increased content level of cinnamic acid derivatives, such as p-coumaric acid, caffeic acid, and ferulic and chlorogenic acids; vanillin, syringic, and coniferyl aldehyde; and flavonoids (kaempferol, luteolin, and apigenin) in different proportions. That is why these oil emulsions might be useful as a basis for biomedical products that actively protect cells against degeneration and inflammation [37,49]. One of the oils was previously successfully used as supplementation of a wound-healing regimen based on flax fabric [50]. Dressings made from genetically modified flax were a huge success in healing wounds. It was shown that the most beneficial properties are those of dressings made of fabrics derived from modified MB linen. The effectiveness of flax dressings has been confirmed by studies and clinical tests that have presented a reduction or disappearance of wounds [49,51,52]. After applying the dressings, the extent of the lesions decreased significantly, and the pain decreased in almost all patients. The best results of the drug were seen in patients with less extensive ulcers [50,53].

Subsequent research work led to the production of linseed oils from transgenic M and B plant varieties, which were tested for potential chemopreventive use in colorectal cancer [35]. The same oil emulsions M, B, MB, and NIKE were tested in this project in the context of wound healing and protection against oxidative stress on model skin cells [37]. M oil contained mainly polyphenolic compounds with the dominant content of vanillin and vanillic acid, as well as β-sitosterol. However, oil B was characterized by a lower content of polyphenolic compounds, which was also compared to linseed oil from the traditional variety, and an increased amount of oleic acid (OA) and linoleic acid (LA), as well as stigmasterol. MB oil is a mixture of M and B oils in a 1:1 ratio. The contents of fatty acids such as palmitic acid (C16:0) and stearic acid (C18:0), oleic acid (OA, C18:1 n-9), linoleic acid (LA, C18:2 n-6), α-linolenic acid (ALA, C18:3 n-3), saturated fatty acids (SFA), monounsaturated acids (MUFA), polyunsaturated acids (PUFA), and phytosterols such as campesterol, stigmasterol, obustifoliol, β-Sitosterol, Δ5-Avenasterol, cycloartenol, and methylenecycloartanol were determined in the tested linseed oils. Moreover, the content of compounds with antioxidant properties, such as tocopherols (α-Tocopherol, γ-Tocopherol, δ-Tocopherol), Plastochromanol-8, carotenoids (all-trans-β-Carotene, all-trans-Lutein + all-trans-Zeaxanthin, all-trans-Neoxanthin, all-trans-β-Cryptoxanthin), and polyphenols (vanillin acid, vanillin, p-Coumaric acid, syringaldehyde, ferulic acid, coniferyl aldehyde, o-Coumaric acid) [35].

NIKE oil made from a traditional flax variety was characterized by a higher content of palmitic and stearic acids than M, B, and MB oils from transgenic flax varieties. Stearic acid creates a moisture barrier on the skin, enhancing formula texture and stabilization. It also possibly has anticancer and anti-inflammatory activity, which would help calm sensitive skin. [54]. However, palmitic acid is reportedly involved in inflammatory processes in some pathological skin conditions, such as psoriasis. Palmitic acid indirectly stimulates epidermal keratinocytes in psoriasis [55] and could create an inflammatory milieu in the skin by activating both macrophages and keratinocytes [56]. However, fatty acids are common raw materials in cosmetics and also are an important component of skin surface lipids, and their composition and amount affect the skin condition. They can be used as emulsifiers, softeners, or brighteners [57]. Squalene, palmitic acid, stearic acid, and OA augmented the secretion of IL-1β, even in the absence of *propionibacterium acnes*, whereas OA had a selective effect of inducing IL-1β but downregulating IL-6 and tumor necrosis factor TNF-α secretion [58].

Oil B from the transgenic flax variety contained more OA and LA compared to other varieties of transgenic flax, such as M oil and traditional NIKE oil. OA is also a component of skin lipids, as well as a commonly known agent that increases skin penetration, which allows more drugs to enter the skin [59]. LA is particularly important for skin health because it contributes to the formation of essential ceramides that ensure the appropriate structure of the epidermal barrier [60] and is crucial in the angiogenesis process [61]. LA had an anti-inflammatory effect in *P. acnes*-activated macrophages, inhibiting the secretion of interleukin IL-1β, IL-6, and TNF-α [58]. LA facilitates wound healing via the promotion of skin hydration and enhancement of the migration of neutrophils and keratinocytes [62].

Both LA and OA accelerate the development of the skin’s lipid barrier, but LA is a 10-times-more potent activator of PPARα, which is involved in the regulation of keratinocyte proliferation, inflammation, and lipid barrier homeostasis [43]. Also, the most abundant PUFA in human skin is the LA [50]. PUFA content is the same in oil from the traditional NIKE flax variety and M transgenic flax oil. PUFAs, such as LA and OA, modulate the inflammation in the wound and enhance reparative response in vivo [63,64,65] (Table 2). PUFAs can be used to prevent skin diseases but also treat the most common chronic inflammatory diseases, including inflammatory skin diseases such as atopic dermatitis, psoriasis, and acne [19]. Other research groups, testing plant oils in vitro for use on skin wounds, observed that PUFAs influence the production of inflammatory mediators and stimulate the proliferation of epithelial cells. ALA, eicosapentanoic, γ-linolenic, and arachidonic acids significantly increase cell migration in a wound-healing model [43,66]. A summary of studies on the pharmacological effects of in vitro and in vivo models of skin effects of most important active compounds also contained in linseed oils is included in Table 2 below.

The highest amount of ALA was found in NIKE oil. Many studies have shown that ALA exerts significant anticancer effects against breast, prostate, colorectal, hepato-cellular, and pancreatic cancers. ALA has various anticancer effects, including the in-hibition of proliferation, apoptosis, tumor metastases, and angiogenesis, and it has an-tioxidant properties. ALA inhibits cell proliferation by regulating the AMPK/S6 axis and promoting cell apoptosis by directly increasing intracellular lipid peroxidation (LPO) or indirectly reducing the accumulation of NO. The anti-inflammatory effects of ALA may be mediated by blocking the TLR4/MyD88/NF-κB cascade [67,68,69,70,71,72]. ALA also reduces the pathological phenotype of psoriasis by normalizing keratinocyte prolifera-tion and differentiation in vitro [73]. The effects of ALA in cutaneous models have been studied primarily in psoriasis [73,74], as presented in Table 2. Both LA and ALA have been shown to lighten skin following UV-induced hyperpigmentation of the skin in guinea pigs, which was believed to be due to the suppression of melanin production and enhanced desquamation of the pigment from the epidermis [75,76]. The UVB-induced erythema score was significantly lower in mice with topically applied creams containing LA and ALA than in mice with basal cream [77].

NIKE oil also contains the most phytosterols. Its anti-inflammatory properties effect phytosterols, causing a decrease in the production of anti-inflammatory cytokines, reducing the release of inflammatory mediators, and inhibiting inducible nitric oxide synthase (iNOS) and cyclooxygenase-2 (COX-2) [78]. Furthermore, phytosterols have anticancer and antioxidant effects [52]. However, most of the phytosterols—stigmasterol—are found in B oil (42.4 ± 1.3 mg/100 g). Stigmasterol had significant chemopreventive effects in an experimental model of skin cancer that may result from reduction oxidative stress and antigenotoxic properties [79]. The pro-apoptotic effect of stigmasterol on melanoma cells is caused by downregulating the ROS [80]. As shown in Table 2, stigmasterol also has significant anti-inflammatory effects within in vitro [81] and in vivo [82] models.

β-sitosterol, found in the largest amount in M oil (296.6 ± 7.5 mg/100 g), has been shown to be effective in the alleviation of atopic dermatitis in NC/Nga—mouse model of atopic dermatitis. Treatment with β-sitosterol resulted in a reduction in the amount of inflammation-related mRNA and protein in atopic dermatitis lesions and in the levels of histamine, IgE, and interleukin-4 in the serum [82,83]. Other research groups have demonstrated β-sitosterol’s anti-inflammatory effects in various models of dermatitis [81,82], protecting against skin ageing [84], inducing hair growth in the alopecia model [85], and promoting anticancer effects against melanoma [86] (Table 2).

NIKE oil has the most antioxidant ingredients—tocopherols (354.67 ± 3.75 mg/kg), carotenoids (25.03 ± 0.24 mg/kg), and M oil—and polyphenols (308.77 ± 34.79 µg/kg), which showed a gene-protective effect in the entire range of tested concentrations. Most of all, the ingredients are polyphenols—vanillin, especially in M oil (145.91 ± 26.94 µg/kg) and vanillic acid (58.68 ± 5.00 µg/kg). Tocopherols are well-known antioxidants, and the moisturizing agent [87] α-Tocopherol (Vitamin E) plays a crucial role in lipophilic antioxidants, which prevent lipid peroxidation of the cell membrane. Tocopherols also regulate signaling pathways, such as inflammation, cell apoptosis, and proliferation [88,89,90]. Vitamin E lowers the production of inflammatory mediators such as prostaglandin E2 (PGE2) and pro-inflammatory cytokines [90,91], while α-tocoferol protects cutaneous tissues against oxidative damage induced by UV irradiation by antioxidant activity [92,93,94] (Table 2). Carotenoids have a protective effect against oxidative damage by inhibiting the decline in antioxidant enzyme levels in UVA-exposed cells, reducing the levels of UVB-induced oxidative stress metabolites [95]. β-carotene can act as a powerful antioxidant and has anti-inflammatory activity, as was described in Table 2 [96,97]. β-carotene helps in wound healing by triggering an immune response, increasing the number of monocytes and macrophages during inflammation, and its antioxidant properties help regenerate burned skin, which is characterized by intense production of free radicals. The encapsulated β-carotene in a novel gelatin/polyglyceryl stearate/graphene oxide hydrogel was evaluated by the synergistic performance of all four materials for burn woundhealing applications [98].

The impact of polyphenols on the skin is mostly dictated by their physicochemical, anti-inflammatory, immunomodulatory, antioxidant properties, and DNA repair activities, which can be exploited for the prevention of a variety of skin disorders. Many skin diseases, including psoriasis, vitiligo, skin photodamage, atopic dermatitis, and skin cancer, are caused by oxidative stress, which damages DNA, proteins, and lipids [99].

The largest number of polyphenolic compounds, such as vanilin acid (VA), vanillin, o-cumaric acid, syringaldehyde, and coniferyl aldehyde, was present in M oil, with a significant predominance compared to oils B and NIKE. VA exhibits a range of physiological functions, including anti-inflammatory, antimicrobial, antioxidant, and anticancer activities. VA may have potential therapeutic benefits in wound healing by promoting keratinocyte proliferation, migration, and angiogenesis [100,101,102]. Vanillin has the ability to inhibit ROS-induced cell death and causes increase in cell migration, suggesting that vanillin has the ability to heal wounds in vitro [103,104] and anti-melanoma activity [105] (Table 2).

Coniferyl aldehyde (CA) significantly reduced inflammatory reactions in macrophages and keratinocytes mediated by a significant selective inhibition of JAK2-STAT1-iNOS signaling [106] (Table 2).

Although NIKE oil was dominated by two polyphenolic compounds, such as para-coumaric acid (p-CA) and ferulic acid, p-CA possesses various bioactivities such as antioxidant, anti-inflammatory, anticancer, and anti-melanogenic properties by impairing the melanin synthesis pathway. p-CA also reduces ROS production through its antioxidant activity and reduces oxidative stress [107]. p-CA attenuates the inflammation and pigmentation due to strong UVB exposure [108]. Ferulic acid possesses anti-inflammatory, antioxidant, antimicrobial, and anticancer activity against breast, colon, and skin cancer. Ferulic acid is a free-radical scavenger but also an inhibitor of enzymes that catalyze free-radical generation and an enhancer of scavenger enzyme activity. Ferulic acid has a protective role for the main skin structures: keratinocytes, fibroblasts, and collagen, elastin. It inhibits melanogenesis, enhances angiogenesis, and accelerates wound healing [109,110,111,112,113,114]. 

**Table 2:** Summary studies of pharmacological activity in vitro and in vivo models of action on skin of active compounds, which also linseed oils also contain.

Bioactive Compound	Pharmacological Activity	Type of the Study	Study Model	References
OA	Wound healing Anti-inflammatory	In vitro	Mouse embryo fibroblasts Balb/c	Cardoso C.R. et al., 2011 [63]
		In vivo	Balb/c mice	
	Wound healing	In vivo	Wistar rats	Pereira L.K.M. et al., 2008 [64]
LA	Wound healing	In vitro	Human Keratinocytes HK, Human Fibroblast HF	Zhao D. et al., 2022 [65]
		In vivo	Minipigs	
	Wound healing	In vivo	Wistar rats	Pereira L.M. et al., 2008 [64]
ALA	Reduce the proliferation and restore the differentiation of psoriatic KCs	In vitro	Psoriatic fibroblasts and KCs from human patients with psoriasis—tissue-engineered reconstructed skin substitutes	Simarad M. et. al. 2021 [73]
	Anti-inflammatory	In vitro	Psoriatic skin model produced with the addition of activated T cells—biopsis from donors	Morin S. et al., 2022 [74]
Stigmasterol	Antioxidant Anti-inflammatory	In vitro	Human immortalized Keratinocyte cell line HaCaT	Miya G.M. et al., 2023 [81]
	Anticancer	In vitro	Murine melanoma B16F10, Human melanoma A375	Han N.R. et al., 2024 [80]
	Anti-inflammatory	In vivo	Swiss mice	Milani G.B. et al., 2024 [82]
	Chemopreventive skin cancer	In vivo	Swiss albino mice	Ali H. et al., 2015 [79]
β-sitosterol	Antioxidant Anti-inflammatory	In vitro	Human immortal-ized Keratinocyte cell line HaCaT	Miya G.M. et al., 2023 [81]
	Protective against skin aging	In vitro	Human Dermal Fibroblast HDF, Human immortal-ized Keratinocyte cell line HaCaT	Yo H. et al., 2019 [84]
	Anti-inflammatory	In vivo	NC/Nga mice	Han N.R. et al., 2024 [83]
	Anti-inflammatory	In vivo	Swiss mice	Milani G.B. et al., 2019 [82]
	Induce hair growth in alopecia	In vivo	Wistar rats	Prabahar K. et al., 2023 [85]
	Anticancer	In vivo	B16F10 melanoma transplantation to C57BL mice	Iyer D. et al., 2014 [86]
α-Tocopherol	Photoprotection Antioxidant	In vitro	Human immortal-ized Keratinocyte cell line HaCaT	Saleh M.M. et al., 2021 [92]
	Antioxidant	In vitro	Primary mouse epidermal keratinocytes PMK, Cell line from adult BALB/c mouse skin Normal Mouse Keratinocytes isotated form newborn Balb/c mouse	Maalouf S. et al., 2002 [93]
	Antioxidant	In vitro	Human primary fibroblast (HPFs) isolated from perilesional skin of nonmelanoma skin cancer patients_	Camillo L. et al., 2022 [94]
β-caroten	Antioxidant	In vitro	Normal Human Dermal Fibroblasts FEK4	Trekli M.C. et al., 2003 [96]
	Anti-inflammatory	In vivo	Hairless mice HR1	Kake T. et al., 2019 [97]
Vanillin	Wound healing	In vitro	Human immortal-ized Keratinocyte cell line HaCaT, Primary Skin Fibroblasts	Sinsuebpol C. et al., 2023 [103]
	Anticancer	In vitro	B16F10 cell line	Pourhadi M. et al., 2022 [105]
		In vivo	C57BL6 mice injected murine melanoma B16F10	
Vanillin acid	Wound healing	In vitro	Human immortal-ized Keratinocyte cell line HaCaT	Zhu X. et al., 2024 [100]
Coniferyl aldehyde	Anti-inflammatory	In vitro	Murine macrophages RAW264.7, Human immortal-ized Keratinocyte cell line HaCaT	Akram M. et al 2016 [106]
		In vivo	Sprague Dawley rats	
p-Coumaric acid	Hypopigmenting	In vitro	Human epidermal melanocytes HEMs	Song K. et al., 2011 [108]
		Ex vivo	Porcine skin	
Feluric acid	Antioxidant	In vitro	Human immortal-ized Keratinocyte cell line HaCaT	Pluemsamran T. et al., 2012 [110]
	Antioxidant	In vitro	Normal Human Dermal Fibroblasts NHDF	Hahn H.J. et al 2016 [111]
	Antioxidant	In vitro	Human Dermal Fibroblast adult HDFa	Nagarajan R. et al 2014 [112]
	Antioxidant	In vitro	Neonatal Normal Human Dermal Fibroblasts HDFn	Calabrese V. et al., 2008 [113]
	Wound healing	In vivo	Wistar rats	Ghaisasm M. et al., 2014 [114]

Furthermore, the appropriate concentration of linseed oil is important in ensuring appropriate wound-healing conditions. In the study, in which semi-solid preparations of linseed oil (1% or 5%) were administered to skin wounds in rats, 100% reepithelialization was found on day 14, while in the Vaseline control group, it was only 33.33%. The therapeutic potential of linseed oil in the regeneration process of damaged skin is maintained only when used in concentrations (1% and 5%); a higher concentration of 10% does not ensure effectiveness [115]. In this study, the final concentrations of the chemicals in emulsion were 1% lecithin, 2.5% flax oil, 2.5% Tween 80, and 2.5% glycerol [35,37]. The same ingredients and their proportions were previously used to prepare W92, W86, and GT oil emulsions [37]. Linseed oils with appropriate composition can serve as potential drug carriers that will deliver medicinal substances to the skin [116]. All ingredients used to prepare linseed emulsions are safe for human skin and are often used to prepare drug formulations for use on the skin [117,118,119,120].

These studies assessed the safety of emulsions based on M, B, and MB oils obtained from transgenic flax seeds and non-transgenic NIKE flax seeds in a cytotoxicity test against Chinese hamster lung fibroblasts (V79-4), normal human dermal fibroblasts (NHDF), human skin microvascular endothelial cells (HMVEC), normal human epidermal keratinocytes (NHEK), and epidermoid carcinoma tumor lines (A431). Antioxidant and gene-protective properties were identified for the tested oil emulsions in a healthy cell model and in an in vitro model of cells under oxidative stress. The wound-healing regenerative potential of these linseed emulsions was also assessed in the proliferation, cell cycle, migration, and apoptosis and necrosis assays.

All tested oil emulsions do not show cytotoxicity towards NHDF, HMVEC, and NHEK cell lines, while B and MB emulsions also do not show cytotoxicity towards V79-4 and THP1 lines. Emulsion B has the strongest antiproliferative properties against the A431 cells. At the same time, in the comet test, only this oil emulsion showed statistically significant DNA damage in V79-4 cells, but the lowest concentration of this oil emulsion caused a gene-protective effect. The strongest antiproliferative effect on A431 cells, linseed emulsion B, may be due to the presence of the most amount of stigmasterol with anticancer properties. The probable mechanism of action of linseed emulsion B is the activation of LXR in A431 cancer cells [121].

B and MB oil emulsions induced the highest proliferation of V79-4 cells. Figure 1 presented the influence of the tested linseed emulsions on the cell cycle with the predominance of the G0/G1 phase, and only for the MB oil emulsion is there a slight predominance of the S phase. In the migration test, the highest degree of migration of V79-4 cells was observed for the B linseed emulsion, which confirms the results of the proliferation test. For linseed emulsion B, most cells occurred in the G0/G1 phase. In studies on the HMVEC cell line in the proliferation test, all results were significantly different from the control. The highest proliferation occurred for MB oil emulsion. Also, for this oil emulsion, most cells occurred in the S phase. The largest number of NHEK cells in the S phase occurred in oil emulsion B, for which cell proliferation was at the control level. In studies on the NHEK cell line in the proliferation test, the highest proliferation also occurred for the MB oil emulsion, but for this oil emulsion, the largest number of cells occurred in the G0/G1 phase. After division, most cells of the human body enter the G0 phase. Such cells do not undergo further division, and after differentiation, they perform specific functions in tissues. However, a small constant percentage of cells in the G0 phase retain the ability to divide. The return of cells from the G0 phase to the cell cycle enables tissue regeneration by replacing dead or damaged cells with living cells.

The tested oil emulsions are characterized by the ability to eliminate damaged cells. This is indicated by the presented studies on apoptosis and necrosis of V79-4 cells. Oil emulsions strongly induced apoptosis in damaged V79-4 cells. The lowest number of cells in the apoptosis phase was observed in cultures treated with NIKE emulsion. A statistically significant increase in the level of apoptosis occurred for linseed emulsion M at two concentrations: 1 mg/mL and 2.5 mg/mL, and for emulsions B and MB at a concentration of 2.5 mg/mL. Necrosis is statistically significant only for oil emulsion B, with a concentration of 0.25 mg/mL. The process of apoptosis in cells definitely dominates over the process of necrosis. Apoptosis is an important process involved in the early phases of wound healing. In normal wound healing, programmed cell death is necessary for removing inflammatory cells and afterwards for scar formation. This removal occurs without tissue damage or inflammation [122,123,124,125].

The most statistically significant reduction in the level of free-oxygen radicals occurred for oil emulsion B in V79-4 cells that were not treated with hydrogenium peroxidatum, which indicates the greatest antioxidant effect of this emulsion. In the in vitro model of oxidative stress caused by hydrogenium peroxidatum on V79-4 cells, statistically significant antioxidant effects of the tested emulsions from transgenic oils M, B, and MB were obtained, but also in this case, it was shown that the strongest antioxidant properties were demonstrated by linseed emulsion B. The results are surprisingly sufficient: M linseed oil is characterized by the highest content of polyphenolic compounds, especially vanillic acid and vanillin, but despite this, the antioxidant capacity of this oil is weaker than oil B, which is characterized by an increased content of stigmasterol, a polysterol, compared to other tested oil emulsions. Stigmasterol is known for its strong oxidative stress-reducing properties [78,80,126,127,128]. In this experiment, it was shown that stigmasterol has stronger antioxidant properties—reducing oxidative stress—than a mixture of synergistically acting polyphenol compounds.

The remaining oil emulsions M and MB, as well as NIKE, showed a gene-protective effect in the entire range of tested concentrations. All oil emulsions from transgenic flax varieties resulted in a significant reduction in intracellular ROS formation in cultures exposed to H_2_O_2_ compared to controls. Oils from transgenic flax varieties reduced the level of free radicals the most. The greatest reduction in the level of free radicals occurred for oil B. The protective effect of wound healing in the case of the tested oil emulsions can be achieved through a direct antioxidant effect, where a significant reduction in the number of free radicals allows for enhanced repair of DNA damage, and a pro-apoptotic effect is activated against damaged cells. The exposure of V79-4 cells to the tested linseed emulsions significantly increases the resistance of these cells to oxidative stress. The V79-4 cell line is often used in genetic toxicology studies, including tests examining cytotoxicity, mutagenesis, DNA damage, and repair mechanisms. V79-4 cells are characterized by a stable karyotype, shortened cell cycle, fibroblast-like morphology, and rapid growth rate, making the V79-4 cell line an ideal tool for experiments requiring consistent and reproducible results in mammalian cell models. V79-4 cells are a widely recognized model in research fields, including cancer biology, pharmacology, and environmental toxicology. Therefore, this cell line was selected to determine the safest action of the tested linseed emulsions [129].

The results of the conducted research indicate that oil emulsion B has the most appropriate regenerative properties, for which the highest migration of V79-4 cells was achieved in the scratch test. Compared to the other tested linseed emulsions, oil emulsion B is characterized by the highest content of OA, which increases the penetration of the active ingredients of linseed oil into the cells and LA, which increases wound healing by optimally moisturizing the wound and intensifying cell migration. However, too much ALA content in linseed oil, as in NIKE and M oils, significantly decreases cell apoptosis, which is necessary in the regeneration process to replace dead or damaged cells with living cells. Perhaps limited penetration into cells by insufficient OA and LA content inhibits cells proliferation. It is probable that too many anti-inflammatory compounds, such as carotenoids and tocopherols in NIKE oil and dominant polyphenol compounds in M oil, inhibit the wound-healing process. The use of linseed emulsion with an excessively strong anti-inflammatory effect may limit the transition of the wound-healing process from the homeostasis phase to the inflammation phase, which leads to the migration of immune system cells to the wound site, and then the proliferation phase of wound healing begins.

Since chronic wounds often occur under chronic inflammatory conditions, normal wound repair proceeds through four overlapping sequential steps: hemostasis, inflammation, proliferation, and, finally, maturation/remodeling. In response to an injury, the hemostasis phase of wound healing begins. The inflammation phase is when degraded collagen fragments and local hypoxia cause nearby immune cells, such as macrophages, to migrate to the wound site. The proliferation step of wound healing caused the activation of profibrotic activity by fibroblasts and keratinocytes. The final step of wound healing occurs when fibroblasts remodel the ECM by realigning and depositing collagen fiber such that collagen is matured into complex structures [130]. Inflammation is an essential and critical stage of wound healing, but it has two sides. Appropriate inflammation promotes wound healing by clearing pathogens and removing necrotic cellular debris, etc., whereas prolonged inflammation would lead to excessively high levels of inflammatory cytokines and high protein hydrolyzing enzyme activity, which can disrupt tissue repair and, in turn, result in the emergence of chronic wounds [131].

ALA, LO, and OA contained in linseed oil, as well as phenolic compounds, have antibacterial properties [14,132]. Long-term inflammation and bacterial infection can inhibit the healing process and lead to scar formation. The excessive ROS produced at the inflammation stage induces oxidative stress, lipid peroxidation, and severe damage to cells, thereby delaying the transformation of the inflammation stage to the proliferation stage [133]. Effective wound care remains a significant challenge due to the need for infection prevention and inflammation reduction. Linseed oil B contains large amounts of biologically active compounds, such as unsaturated fatty acids (mainly ALA, OA and LA) and M oil—polyphenols, which have antibacterial properties [14,132].

Akl et al. demonstrated antibacterial activity and that flax seed extract was effective against only four of seven strains of bacteria. [134]. Flaxseed nanoemulsion showed anti-inflammatory and antibacterial activity by inhibiting *Staphylococcus aureus* and *Pseudomonas tolaasii* [135]. In addition, seed oil has good antibacterial activity against *E. coli* and *S.aureus* [136]. The in vitro antimicrobial activity of the linseed oil against *Staphylococcus aureus*, *Streptococcus agalactiae,* and *Escherichia coli* was comparable to that of cefoperazone, while the antimicrobial activity against *Enterococcus faecalis*, *Micrococcus luteus,* and *Candida albicans* was greater than that of cefoperazone [137]. Linseed oil synergizes the antimicrobial potential of gemifloxacin when used simultaneously in various combinations [138]. Linseed oil has antibacterial properties and has also presented synergism with boric acid, which is successfully used in the treatment of wounds. Omega fatty acids and LA contained in linseed oil support healing skin wounds and reduce inflammation/itching [48,139,140]. Additionally, linseed oil has significant antifungal activity against *Aspergillus versicolor*, *A. niger* and *Penicillium verrucosum* [141], and *Candida albicans* [142]. Therefore, linseed oil B will also be a suitable base for medicines with antibacterial and antifungal effects on the skin, which may enhance their effect.

*S. aureus*, a common inhabitant of the human microbiota, is usually found on the skin surface where they coexist with other microbes such as *Actinobacteria, Firmicutes, Bacteroidetes,* and *Proteobacteria*. Most patients exhibit atopic dermatitis flare, and the results are excessive in staphylococcal colonization [143]. Previous studies have shown that topical and systemic anti-inflammatory treatments reduce *S aureus* colonization in patients with atopic dermatitis [144]. This research project proposed an innovative use of transgenic linseed oils for inflammatory skin diseases such as atopic dermatitis or psoriasis, which are closely related to inflammation and oxidative stress [145]. The conducted research showed that the tested transgenic oil emulsions are safe for human skin because they do not induce the proliferation of skin cancer cells and, at the same time, induce the migration processes of normal human skin cells. Additionally, their use increases the ability to eliminate damaged cells. Moreover, the balanced composition of active compounds in transgenic oils ensures appropriate synergism of the ingredients’ action, which provides them a gene-protective effect and an increased antioxidant effect, resulting in increased protection of skin cells against oxidative stress, which plays an important role in the pathogenesis of atopic dermatitis and psoriasis [146,147]. However, the compound with the strongest antioxidant effect compared to synergistic mixtures of polyphenolic compounds is stigmasterol. The greatest composition of oil B is likely the combination of stigmasterol with polyphenolic compounds in appropriate concentrations, which allows for achieving a synergistic effect in the regeneration process of cells exposed to oxidative stress. Additionally, emulsions created using linseed oils are an attractive delivery system in the field of dermatology due to their ability to improve the drug-release profile and skin penetration [145]. For this reason, the tested linseed emulsions are, at the same time, a ready-made drug formulation for administration to skin inflammation or healing wounds, but they will also be an appropriate carrier of antibacterial or antifungal drugs for infected skin.

The limitation of the in vitro experiments is the lack of testing in an in vivo model. In vivo studies are strictly regulated and, where possible, replaced with alternative methods such as cell culture studies. By using the in vitro model at this stage of research, the number of animals used in future experiments can be significantly reduced. All experiments were conducted with a test power of at least 80% [144,145,146]. At the same time, linseed oils used to prepare the tested emulsions come from genetically modified plants, i.e., genetically modified organisms (GMO). By definition (Journal of Laws 2001, No. 76, item 811), GMOs are a biological entity (excluding humans) in which the genetic material has been changed in a way that does not occur under natural conditions. They arise as a result of crossbreeding or natural genetic recombination. Genetically modified (GM) plants and seeds have a fragment of genetic information incorporated into their genome, obtained from another organism, the so-called transgene. Its introduction allows for the quick and precise obtainment of material with the desired characteristics, such as herbicide resistance. All of Poland declared itself a GMO-free zone. In Poland, the basic legal act regulating GMO matters is the Act of 22 June 2001, on genetically modified organisms. Noteworthy here is the prohibition on entering varieties of GMO plants to the national register and the prohibition of admission in Poland for the marketing of genetically modified material sowing. Although it is difficult to question the possible benefits resulting from the use of GMOs, it must be remembered that their widespread use also raises many doubts. The biggest fears are the potential effects of releasing GMOs into the natural environment. The use of GMOs in industry, agriculture, medicine, and environmental protection carries many benefits but also many threats, which makes scientists seriously divided on this issue [148,149,150]. In this situation, in Polish conditions, a safer solution seems to be to obtain linseed oils with wound-healing properties that are used in inflammatory skin diseases by adding appropriate active compounds to the registered raw material to reflect the composition of oil B with the best regenerative properties, or those obtained by other biotechnological in vitro culture techniques. More advanced molecular studies of the mechanism of action of stigmasterol in protection against oxidative stress and the assessment of the tested oil emulsion B in comparison with selected active compounds include: ALA and stigmasterol contained in the TLR4/MyD88/NF-κB cascade responsible for maintaining chronic inflammation. Extended in vitro tests are planned on 3D skin organoids and permeation tests on skin fragments of animal origin. Then, pre-clinical (in vivo) tests are planned in mice with inflammatory skin diseases, such as atopic dermatitis, as well as clinical studies in the veterinary area on dogs and the medical area on human suffering from inflammatory skin diseases, especially atopic dermatitis, in which wounds are formed during the inflammatory process. Also, it is planned to determine the antibacterial activity of the tested linseed oils from transgenic varieties in comparison to linseed oils from traditional varieties and to select the oil with the best properties, which may allow for the expansion of indications for the use of transgenic linseed oils.

## 5. Conclusions

Considering the increasing incidence of chronic wounds and severe wound infections, effective drug delivery to wounded skin is of high importance. The rational development of novel therapeutic systems requires appropriate in vitro testing methodologies [151]. Based on the conducted research, it was shown that emulsions from transgenic oils have gene-protective properties against the tested human epidermis cell lines and protect against irritation and damage, thus promoting wound-healing processes. Moreover, oil emulsions based on transgenic linseed oil (M, B, MB) are effective in antioxidant protection of the skin. The advantages of the tested oil emulsions for wound-healing processes include their non-stick properties and maintaining a moist wound environment, which is crucial for optimal healing. Thanks to the PUFA content, the tested oil emulsions can also increase the skin’s absorption of the medicinal substances they carry. The tested linseeds oil emulsions have hypoallergenic and anti-inflammatory properties, which is why they protect the skin against irritation and have a soothing effect. Therefore, they can be used to treat skin lesions characterized by chronic inflammation, such as atopic dermatitis or psoriasis. The innovation of the conducted research is the proposal of a new indication for the use of transgenic linseed oils, especially B oil, in inflammatory skin diseases whose pathomechanism is based on oxidative stress. In addition, due to the known antibacterial properties of linseed oil, they can be used as a drug or as a component of drugs with antibacterial and antifungal properties. Based on the conducted research, it was determined that linen emulsion B has the best regenerative and protective properties against human epidermis cancer, which is probably due to the presence of an increased amount of stigmasterol in its composition, along with the appropriate content of polyphenol compounds, as well as an increased amount of OA and LA.

## Figures and Tables

**Table 1:** The emulsions of linseed oils on cellular vitality of cancer lines in the MTT assay.

Cell Line	IC_50_ [mg/mL]			
	Linseed Oil Emulsions			
	NIKE	M	B	MB
NHDF	NA	NA	NA	NA
HMVEC	NA	NA	NA	NA
NHEK	NA	NA	NA	NA
V79-4	4.79 ± 0.33	5.48 ± 0.28	NA	NA
THP1	2.82 ± 0.50	2.25 ± 0.52	NA	NA
A431	3.21 ± 0.25	2.39 ± 0.77	1.90 ± 0.26	NA

NA—no cytotoxicity was determined at the tested concentration range 0.1–2.5 mg/mL.

## Data Availability

Data availability statements are available after contact with corresponding author: izabela.jeskowiak-kossakowska@umw.edu.pl.
